# Developing a competency model for Chinese general practitioners: a mixed-methods study

**DOI:** 10.1186/s12960-024-00912-1

**Published:** 2024-05-27

**Authors:** Xue Gong, Xu Zhang, Xinyan Zhang, Yixuan Li, Yang Zhang, Xiaosong Yu

**Affiliations:** 1 Library of China Medical University, Shenyang, China; 2 Department of Science and Education, Chaoyang Central Hospital, Chaoyang, China; 3 School of Public Health, Jinzhou Medical University, Jinzhou, China; 4 Department of General Practice, the First Hospital of China Medical University, Shenyang, China; 5 Education Centre for Clinical Skills Practice of China Medical University, Shenyang, China

**Keywords:** General practitioner, Competency, China, Assessment

## Abstract

**Background:**

The Chinese government has formulated a series of policies and strengthened training of general practitioners (GPs) to support their role as “gatekeepers” of residents’ health. This study aimed to explore the core competencies of Chinese GPs and develop a competency framework in line with China’s actual conditions, which can provide a more scientific basis for the education, training, and evaluation of GPs.

**Methods:**

Literature analysis and behaviour event interviews were conducted to build the competency dictionary and the initial version of the competency model. Two rounds of Delphi were performed to gain consensus on the final model. The questionnaire survey was carried out in 10 provinces (municipalities, autonomous regions) of China, and GPs were invited to score the importance of each competency item. The total sample was randomly divided into two groups. One group was for exploratory factor analysis (EFA), and the other was for confirmatory factor analysis (CFA) to examine the scale’s reliability and validity.

**Results:**

The dictionary of general practitioners’ competency including 107 competency items was constructed. After two rounds of Delphi, a consensus was reached on 60 competencies in 6 domains. A total of 1917 valid questionnaires were obtained in the nationwide survey. The average importance score of all second-level indicators is 4.53 ± 0.45. The Cronbach’s α coefficient is 0.984. The results of the five factors extracted by EFA showing the 68.16% cumulative explained variance variation is considered to be consistent with the six dimensions obtained by Delphi after thorough discussion. The model fitness indexes obtained by CFA were acceptable (χ^2^/df = 4.909, CFI = 0.869, NFI = 0.841, RMSEA = 0.065). The values of the composite reliability (CR) of the six dimensions were all greater than 0.7 (0.943, 0.927, 0.937, 0.927, 0.943, 0.950), and the average of variance extracted (AVE) were all greater than 0.5 (0.562, 0.613, 0.649, 0.563, 0.626, 0.635). The results showed that the model has good reliability and validity.

**Conclusion:**

A competency model for GPs suited to China has been developed, which may offer guidance for future training and medical licensing examinations of GPs.

## Introduction

Health is a permanent goal pursued by the people. China is the world’s most populous country, and the Chinese government has consistently promoted the high-quality development of medical and health services and has given top priority to improving people’s health. Primary health care has received considerable attention in China since launching a new round of healthcare reform in 2009. The role of primary health care is further emphasised by the “Healthy China 2030 Planning Outline” approved in 2016 [1, 2]. General practitioners (GPs) are medical talents who have received a wide range of medical professional education and training and are often referred to as “health gatekeepers” [3]. As the leading providers of primary health care, sufficient and qualified GPs are critically important for primary health care to reach the goal of every citizen having equal access to affordable health care services. In 2011, the Chinese government formally launched its ambitious plan to establish a general practice system to reach at least two GPs per 10,000 population, a total of 300,000 GPs by 2020 [4]. To achieve this goal, the Chinese government has made many efforts. Government statistics show 2.9 GPs per 10,000 population in 2020 [5]. In 2018, the Chinese government announced a new plan of five GPs per 10,000 population, a net GP workforce of 700,000 GPs by 2030, an additional increase of 300,000 in a decade [6]. According to the survey results, less than 40% of GPs at the grassroots have bachelor’s degrees or higher degrees, and most GPs have not received standardised resident training [7]. Therefore, rapidly training more competent GPs has been regarded as an urgent problem [8]. Then, a fundamental issue that must be addressed is what kind of GPs are competent or what abilities a capable GP should have.

Since the early 2000s, medical education has transformed traditional education into competency-based education [9]. More and more health systems and medical schools worldwide have adopted competency-based medical education [10–12]. The first step to implementing competency-based medical education is to define the competencies physicians should have. Some developed countries have identified the necessary competencies to be a qualified GP or family physician. The College of Family Physicians of Canada (CFPC) designed a competency framework CanMEDS-Family Medicine and described the roles and competencies required during Canadian family physicians’ work [13]. The Royal College of General Practitioners (RCGP) offered the GP curriculum based on the General Medical Council’s (GMC) generic professional capabilities framework and proposed five areas of capability and 13 specific capabilities for general practice [14]. The WONCA Europe released the European Definition of General Practice/Family Medicine 2023 edition, describing the core competencies required for GPs and family physicians [15]. The Accreditation Council for Graduate Medical Education (ACGME) Family Medicine Milestones arranged the elements of physician competencies into levels, and Milestones 2.0 was effective on July 1st, 2020 [16]. The competencies GPs need to possess and the training modes of GPs vary from country to country as they are affected by local, political, social, and economic circumstances. However, so far, China has not yet established a national-level competency evaluation system for GPs that adapts to China’s national conditions. On January 18th, 2018, the National Health Commission of China released the file “National Physician Qualification Examination Development Plan 2018–2020” and put forward two main tasks: researching GPs’ competencies and formulating competency-oriented admittance standards and examination syllabus, respectively. Thus, a set of core competencies suitable for Chinese GPs is expected to be developed.

## Methods

**Fig.1:**
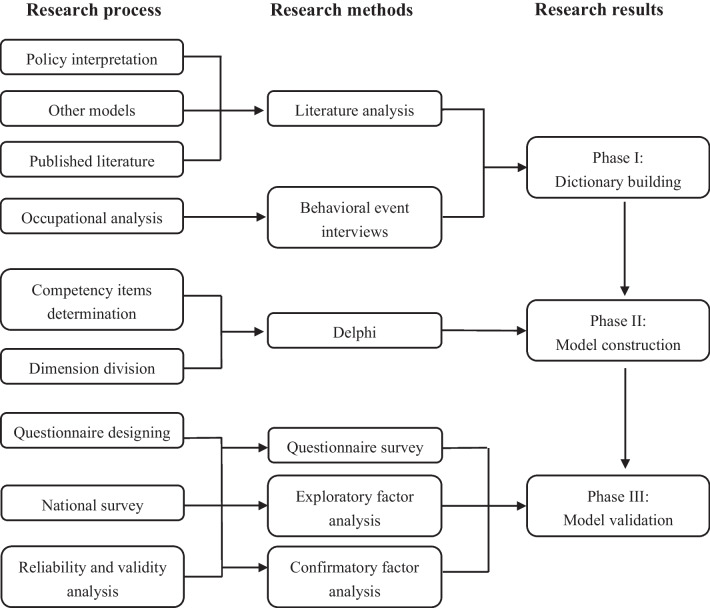
Methodological steps used in this study

This study was conducted from January 2019 to August 2021. The general competency model for Chinese GPs was developed in three phases: building the competency dictionary, constructing the competency model, and validating the model. Figure 1 shows the technological flow of our research.

### Phase I: building the GPs’ competency dictionary

In the first phase, the methods of literature analysis and behaviour event interviews were used to collect the competency elements and construct the dictionary. The literature was mainly from policy documents, established competency models of GPs from other countries, and bibliography databases. The development direction and trend for GPs’ careers and the abilities required for GPs in China were clarified by interpreting the Chinese policy documents concerning GPs in detail. Mature competency models from other countries were obtained by searching their official websites. We collected the literature from five databases (PubMed, Thomson Reuters Web of Science Core Collection, China National Knowledge Infrastructure, and Wanfang Data) from 2000 to 2018. The search strategies in PubMed and Web of Science were as follows:

PubMed: (general practitioner[mesh] OR family physician[mesh]) AND (competency[tiab] OR competence[tiab] OR competencies[tiab] OR ability[tiab] OR abilities[tiab] OR capability[tiab] OR capabilities[tiab] OR capacity[tiab] OR capacities[tiab] OR skill OR skills).

Web of Science: TS = (general practitioner OR family physician OR family doctor) AND TS = (competency OR competence OR competencies).

Behaviour event interviews (BEIs) were conducted with 22 GPs from different regions of China who had engaged in general practice-related work for five or more years. Half of the GPs were assigned to the high-achievement group, and the other half were in the low-achievement group. High achievers were individuals who had been awarded the national honour or won the national prize in general practice by the end of June 2019. Low achievers were defined as individuals who had not received any national award.

The interviews were semi-structured. The first part is to obtain basic information about the interviewees, such as their routine work. The second part is to collect the most impressive and regretful events during their work and their feelings. The last question asks the interviewees to summarise the competencies they consider necessary for GPs. The interviews were kept under approximately 50 min long. After the interview, two research group members transcribed the recording word by word in a unified format in time. Then, they extracted the competency elements from the text according to the competency dictionary compiled in the previous step.

### Phase II: establishing the competency model

In the second phase, the Delphi technique was adopted to revise the preliminary model and determine the final model. The selection of experts is the key to the successful implementation of Delphi. The experts in this study are required to meet the following conditions: (1) they have long engaged in general practice-related clinical, teaching, or administrative work and have a complete understanding of the development of general practice in China and other countries; (2) most of the experts are current or former members of the General Practice Branch of Chinese Medical Association or other national associations; (3) more experts from the Eastern region of China are selected due to the rapid development of general practice service; (4) they are willing to attend this research. We used email or WeChat to communicate with experts. The Delphi questionnaire was created based on the preliminary model. Experts were asked to rate the importance of competencies on a five-point Likert scale where 1 = “not important at all” and 5 = “very important”. The space was provided for experts to write their suggestions or opinions, such as adding new items. After the questionnaires were returned, we calculated the arithmetic mean, full score ratio, and coefficient variations (CV) of indicators based on expert scores and modified the indicators according to experts’ suggestions. The items with arithmetic mean ≥ 4.00, full score ratio ≥ 0.3, or CV ≤ 0.25 were considered more important and acceptable for entering the next steps in this research. Based on the results of the first round, the next round of consultation was conducted. This survey stopped until experts had consistent attitudes.

### Phase III: validating the competency model

At this stage, a cross-sectional survey was conducted. We recruited Chinese GPs from different-grade medical establishments. All the participants gave written informed consent. They could withdraw from the study at any time and refuse to answer questions for any reason.

The sample size for reliability and validity evaluation was 5–10 times the number of items [17, 18]. Considering some responses might be invalid, 20% of extra questionnaires were added. Thus, a minimum sample size of 660 was necessary for reliable results, as the preliminary instrument has 60 items.

The stratified and convenient sampling methods ensured the sample was representative. This survey was conducted in 10 provinces (autonomous regions and municipalities directly under the Central Government) of China in the Northeast (Jilin, Liaoning), Eastern (Beijing, Shanghai, Jiangsu, Guangdong), Central (Shanxi, Henan), and Western (Guizhou, Shanxi). The surveyors received training to ensure they understood the questionnaire well.

The sample was divided into two random subsamples to prove the factor validity. Exploratory factor analysis (EFA) by principal component analysis applied to the first half of the sample was used to explore the structure and extract common factors of the competency model. The Kaiser–Meyer–Olkin (KMO) and Bartlett’s sphericity test were studied for the suitability of the sample. The selection of factors mainly depends on Kaiser’s rule that whether they had eigenvalues greater than one [19]. Items loaded above 0.35 to the factors were considered valid. Confirmatory factor analysis (CFA) was performed in the second half of the sample to test the accuracy of the composition of each dimension and the fitting degree between the model and the actual survey data obtained from the EFA [20]. Some indices were obtained for assessing the model fitting degree, such as Chi-square (χ^2^)/degrees of freedom (df), comparative fit index (CFI), root-mean-square error of approximation (RMSEA), and normed fit index (NFI). Cronbach’s alpha was calculated for all common factors and dimensions to assess the scale’s internal consistency. The values of composite reliability (CR) and the average of variance extracted (AVE) were also calculated to test the convergent validity.

Data were input with Epidata software version 3.0 and analysed with IBM SPSS Statistics version 23.0 for EFA and AMOS version 24.0 for CFA.

## Results

### Analysis of literature and BEIs

In the first stage, with the search expression, 1493 articles from China National Knowledge Infrastructure, 677 from Wanfang Data, 1668 from PubMed, and 1752 from Web of Science. Twenty-two GPs participated in the interviews. Combined with the results of literature analysis and BEIs, a competency dictionary containing 107 items was finally built. Then, a preliminary competency model for Chinese GPs composed of 6 dimensions (first-level abilities) and 80 items (second-level abilities) was conducted after discussion. The six dimensions were: (a) basic medical services, (b) public health services, (c) information and management ability, (d) medical knowledge and lifelong learning, (e) interpersonal communication and teamwork, and (f) professionalism and personal characteristics.

### Results of two rounds of Delphi

Two rounds of the Delphi consultation were carried out with 39 experts from general practice, health management, and medical education. They all had the senior professional title. Of the 39 experts, 37 had served or served in the official general practice associations.

A total of 42 questionnaires were sent out, 39 valid questionnaires were recovered, and the effective recovery rate was 92.86% in the first round of consultation. Questionnaires for the second round were distributed to experts who responded to the first round. Thirty-five valid questionnaires were collected, with an effective rate of 89.74%. The arithmetic means of 79 (91.86%) competency items were higher than 4, 84 (97.67%) items had a full score ratio higher than 0.30, and the CV of 78 (90.70%) items were lower than 0.25 in the first round. Only 4 (5.80%) items had arithmetic means lower than 4, 5 (7.25%) had a lower full score ratio than 0.3, and 63 (91.30%) had CVs lower than 0.25.

Competencies are defined as work-related knowledge, skills, traits, and motives. After two rounds of Delphi consultation, the competency model comprised six dimensions and 60 items (Table 1). Dimensions A to F were named basic medical services, basic public health services, information utilisation ability and management ability, medical knowledge and lifelong learning, interpersonal communication and teamwork, and professionalism, respectively.

**Table 1:** The competency framework for Chinese GPs and importance score by GPs

Dimensions	Items	Importance score (X ± S)
A. Basic medical services	A_1 Have general practitioners′ clinical thinking	4.56 ± 0.60
	A_2 Collect medical history thoroughly and accurately	4.66 ± 0.53
	A_3 Perform standardised physical examination and pay attention to mental state	4.66 ± 0.53
	A_4 Write the medical documents following the standardisation	4.66 ± 0.54
	A_5 Select the laboratory examination properly and interpret the results correctly	4.65 ± 0.56
	A_6 Be proficient in basic diagnostic procedures	4.65 ± 0.54
	A_7 Be able to diagnose and treat common diseases in communities	4.68 ± 0.53
	A_8 Master first-aid skills	4.65 ± 0.57
	A_9 Identify the patients at acute, severe stages and refer them promptly	4.69 ± 0.53
	A_10 Manage chronic non-communicable diseases and follow up with the patients	4.60 ± 0.58
	A_11 Provide the family-based services	4.47 ± 0.67
	A_12 Use drug rationally	4.68 ± 0.54
	A_13 Participate in hospice care	4.48 ± 0.66
B. Basic public health services	B_1 Be familiar with the knowledge and process of prevention and control of common infectious disease	4.57 ± 0.59
	B_2 Organize community health education	4.50 ± 0.61
	B_3 Offer common psychological consult	4.39 ± 0.72
	B_4 Manage the particular population in the community	4.49 ± 0.63
	B_5 Assist in handling public health emergencies	4.58 ± 0.58
	B_6 Provide community rehabilitation services	4.46 ± 0.66
	B_7 Promote pre-natal and post-natal care	4.39 ± 0.72
	B_8 carry out the diagnosis of the community health state	4.47 ± 0.65
C. Information utilisation ability and management ability	C_1 Be able to retrieve and analyse medical literature	4.36 ± 0.71
	C_2 Apply information technology effectively	4.43 ± 0.67
	C_3 Establish and maintain resident health records	4.47 ± 0.65
	C_4 Understand healthcare policy	4.46 ± 0.65
	C_5 Know about the local cultural background	4.28 ± 0.78
	C_6 Control the patients’ medical expenses scientifically	4.48 ± 0.64
	C_7 Use the limited healthcare resources reasonably	4.49 ± 0.63
	C_8 Possess some teaching ability	4.38 ± 0.72
D. Medical knowledge and lifelong learning	D_1 Master the basic medical knowledge	4.67 ± 0.54
	D_2 Master the clinical medical knowledge	4.69 ± 0.51
	D_3 Be familiar with the knowledge of epidemiology and medical statistics	4.50 ± 0.66
	D_4 Be familiar with the knowledge of evidence-based medicine	4.49 ± 0.67
	D_5 Be familiar with some medical humanities knowledge	4.46 ± 0.67
	D_6 Master one foreign language at least	4.13 ± 0.96
	D_7 Participate in continuing medical education and training projects actively	4.58 ± 0.61
	D_8 Have the ability of creative thinking	4.45 ± 0.67
	D_9 Have critical thinking skills	4.41 ± 0.72
	D_10 Write and publish scientific research papers actively	4.13 ± 0.93
E. Interpersonal communication and teamwork	E_1 Establish a mutually trusted, lasting, and stable doctor–patient relationship	4.58 ± 0.58
	E_2 Respect the local customs of patients	4.47 ± 0.66
	E_3 Protect patient privacy and right to know	4.61 ± 0.58
	E_4 Deal with ethical problems properly	4.55 ± 0.59
	E_5 Prevent and resolve conflicts between doctors and patients	4.61 ± 0.56
	E_6 Convey bad news to patients skillfully	4.49 ± 0.68
	E_7 Make decisions together with patients and their families	4.53 ± 0.62
	E_8 Explain medical information in an easily understandable way	4.59 ± 0.58
	E_9 Work in harmony with colleagues	4.62 ± 0.55
	E_10 Work with other teams effectively	4.61 ± 0.55
F. Professionalism	F_1 Abide the medical rules and regulations strictly	4.66 ± 0.52
	F_2 Have the awareness of basic services	4.60 ± 0.56
	F_3 Be self-sacrificing	4.54 ± 0.61
	F_4 Make good career planning	4.53 ± 0.61
	F_5 Have confidence and objective self-evaluation	4.51 ± 0.62
	F_6 Be loving, sympathetic, and empathetic to patients	4.61 ± 0.54
	F_7 Have the ability to insight	4.60 ± 0.55
	F_8 Have patience and good psychological regulating ability	4.61 ± 0.54
	F_9 Provide fair medical care services	4.60 ± 0.57
	F_10 Wear in good taste fit to doctors’ identity	4.54 ± 0.59
	F_11 Be achievement-oriented in daily work	4.54 ± 0.60

### Results of the reliability and validity of the scale

In total, 2126 questionnaires were gathered, and 1917 were considered valid (90.17%) in the third phase. The quantity meets the basic requirements for the required sample size. Among the effective respondents, 677 were males and 1240 were females. The demographic characteristics of the GPs participating in this survey are shown in Table 2.

**Table 2:** Demographic characteristics of the GPs in this survey

Characteristic	Category	Frequency	Percent (%)
Gender	Male	677	35.32
	Female	1240	64.68
Working units	Community health centre	1200	62.60
	Secondary hospital	67	3.50
	Tertiary hospital	365	19.04
	Township health centre	285	14.86
Professional titles	Primary	639	33.33
	Middle	766	39.96
	Vice-senior	347	18.10
	Senior	95	4.96
	No professional title	70	3.65
Education qualification	Junior college and below	259	13.51
	Bachelor	1294	67.51
	Master	325	16.95
	Doctor	39	2.03
Region	Northeast China	454	23.68
	Eastern China	798	41.63
	Central China	374	19.51
	Western China	291	15.18

The values of the Cronbach’s alpha for the full scale were 0.984, 0.946 for Dimension A, 0.927 for Dimension B, 0.935 for Dimension C, 0.920 for Dimension D, 0.946 for Dimension E, and 0.955 for Dimension F, demonstrating strong internal consistency.

The results of the questionnaire survey show that the average importance score is 4.53 ± 0.45. The average score of each second-level indicator is above 4 (Table 1). According to the scale level, the importance level is “important”, indicating that the indicators were approved by the GPs participating in this survey. In overall ranking, the top three are “A_9 Identify the patients at acute, severe stages and refer them promptly”, “D_2 Master the clinical medical knowledge”, and “A_7 Be able to diagnose and treat common diseases in communities”. “D_6 Master one foreign language at least” and “D_10 Write and publish scientific research papers actively” ranked last.

EFA was performed with the first half of the sample (*N* = 979). The KMO value was 0.982, sufficient to indicate a strong relationship [21]. Bartlett’s sphericity test, χ^2^ = 57,014.459, df = 1770, and *p* < 0.001, showed that the data were suitable for EFA. The eigenvalues were set to be greater than 0.35 when EFA was performed. After principal component extraction, five factors with initial eigenvalues greater than one were obtained, accounting for 68.14% of the overall variances. According to the rotated component matrix results, the items in Dimension A were all concentrated in the second factor, and items in Dimension B were focused on the third factor. Items in Dimension C, except C_3 and C_7, were concentrated in the fourth factor. Four items (D_1、D_2、D_3、D_4) of Dimension D focus on the fifth factor, and five (D_5、D_6、D_8、D_9、D_10) in the fourth factor. Items in E and F were concentrated in the first factor (Table 3).

**Table 3:** Exploratory factor analysis loadings in sample 1 (*N* = 979)

Items	Factors				
	1	2	3	4	5
A_1		0.609			
A_2		0.716			
A_3		0.725			
A_4		0.747			
A_5		0.754			
A_6		0.759			
A_7		0.794			
A_8		0.685			
A_9		0.746			
A_10		0.616			
A_11		0.467			
A_12		0.723			
A_13		0.483			
B_1			0.520		
B_2			0.661		
B_3			0.671		
B_4			0.654		
B_5			0.606		
B_6			0.609		
B_7			0.676		
B_8			0.673		
C_1				0.548	
C_2				0.455	
C_3			0.612		
C_4				0.367	
C_5				0.545	
C_6				0.369	
C_7			0.449		
C_8				0.494	
D_1					0.655
D_2					0.653
D_3					0.454
D_4					0.389
D_5				0.572	
D_6				0.795	
D_7	0.526				
D_8				0.538	
D_9				0.643	
D_10				0.759	
E_1	0.677				
E_2	0.600				
E_3	0.641				
E_4	0.660				
E_5	0.699				
E_6	0.619				
E_7	0.635				
E_8	0.686				
E_9	0.745				
E_10	0.710				
F_1	0.649				
F_2	0.692				
F_3	0.719				
F_4	0.666				
F_5	0.698				
F_6	0.707				
F_7	0.716				
F_8	0.716				
F_9	0.716				
F_10	0.672				
F_11	0.670				

The CFA was done with the second half of the sample (*N* = 938). The result is shown in Table 4, all within the range of acceptance or ideal, indicating that the results fit well with the 6-factor of the scale.

**Table 4:** Fit indices for CFA of the competency model

	χ^2^/df	CFI	GFI	NFI	RMSEA
Ideal	≤ 3	≥ 0.9	≥ 0.9	≥ 0.9	≤ 0.08
Acceptable	≤ 5	≥ 0.8	≥ 0.8	≥ 0.8	≤ 0.1
Result of the study	4.909	0.869	0.725	0.841	0.065

The CR of the six dimensions were 0.943, 0.927, 0.937, 0.927, 0.943, and 0.950, all greater than 0.7, and the AVE were 0.562, 0.613, 0.649, 0.563, 0.626, and 0.635, respectively, all greater than 0.5, showing the scale had good convergence validity [22].

## Discussion

In the twenty-first century, the third wave of medical education reform with competency as the core has been set off around the world, and the reform of training medical talents has been gradually deepened in China. The competency model is an essential reference for designing, implementing, and evaluating competency-based programmes [23]. Compared with some developed countries, China has no national-level competency models for general practitioners, and research in this area is still at the primary stage. For this reason, an available model of the competencies required for Chinese GPs was constructed in this study.

The choice of methods is of primal importance for constructing the competency model. Our study employed quantitative and qualitative methods, including literature analysis, BEIs, Delphi, and questionnaire surveys. These methods supplement and verify each other, ensuring the results’ reliability, accuracy, and comprehension [24]. Through literature analysis, competency items were extracted as the basis for the competency dictionary. BEIs are considered the most scientifically rigorous approach for identifying competencies and are commonly used in many fields for competency studies [25]. The front-line GPs’ perception of the knowledge, skills, and attitudes required within the context of general practice had been explored by BEIs. The Delphi method is a structured and commonly used technique in health professions to collect and screen expert advice and develop a framework for competency [26, 27]. The selection of the expert panel may limit the generalisability of the study. The Delphi panel usually consists of 15–30 experts [28]. A widely representative group of 39 Chinese and international medical experts involved in general practice participated in this study.

EFA and CFA were used to verify the model of competencies. The results of EFA that five common factors were extracted, accounting for 68.16% of the variances, differed from that of Delphi, in which six factors were obtained. On this issue, experts in the field of general practice and medical statistics in our group believed that the items under the four dimensions of basic medical services, basic public health services, information utilisation ability and management ability, medical knowledge, and lifelong learning mainly were the same as the results from Delphi. The items in the other two dimensions of interpersonal communication and teamwork, and professionalism are mixed into one group, and most of the items are the competencies hidden or beneath the surface, according to the Iceberg Model. Our group believed that the six-dimensional framework constructed by Delphi was more explicit than that of the five common factors and more helpful for comparing different models. The results obtained from CFA have reached their respective defined values, indicating this indicator system has good reliability and validity. The results of the importance score showed that the competency model was widely accepted by the GPs participating in this survey from ten Chinese provinces.

A competency model for Chinese GPs has been successfully built based on the abovementioned methods. The model is a multi-dimensional system comprising 60 competencies, structured into six dimensions: basic medical services, basic public health services, information utilisation ability and management ability, medical knowledge and lifelong learning, interpersonal communication and teamwork, and professionalism, respectively.

The first dimension was named basic medical services, on which 13 items were loaded. Although GPs who provide comprehensive and continuous healthcare at the grass-roots level are considered a new type of clinician, their primary and most important role is still that of clinicians [29, 30]. GPs must master basic clinical skills such as collecting medical history and performing physical examinations. The second dimension consisted of 8 items, focusing on the capabilities of basic public health services. GPs are usually the first point of contact with the community and are critical to any public health crisis response [31]. During the COVID-19 pandemic, GPs in China are at the epidemic’s frontline [32]. GPs play an important role in detecting, reporting, and treating suspected and infected patients early. Some experts proposed that medical colleges should incorporate public health thinking into the training of GPs and integrate public health ability into the core abilities of GPs.

The third dimension contained eight items, emphasising the ability of management and information utilisation. Information technology has infiltrated all areas of our lives, studies, and work, as well as the healthcare field [33]. Learning about information technology and developing the competencies of medical information retrieval and utilisation can help GPs better carry out clinical work and scientific research. GPs’ work involves the health management of individuals, families, and communities, so they also need specific management skills. The fourth dimension included ten items, concentrating on medical knowledge and lifelong learning. Sufficient preclinical and clinical medical knowledge is the foundation for providing medical and health services to patients and community residents. With the development of modern medical science, new knowledge and new technology emerge rapidly and continuously. All doctors, including GPs, should possess the ability of lifelong learning to keep up their knowledge and skill sets to deliver excellent patient care [34].

The fifth dimension, interpersonal communication and teamwork, comprised ten items. GPs must effectively communicate and cooperate with patients, their families, and other health professionals. It has reached a consensus among GPs that gaining the trust of residents, patients, and their families and establishing a long-term, stable, cooperative relationship with them is critical to the success of general practice. The sixth dimension is related to professionalism with 11 items. As an invisible feature in the Iceberg model, professionalism has a decisive impact on the competency of GPs. Because of the particularity of medical work, doctors not only need to possess excellent professional and technical skills, but also good professional ethics and literacy.

Comparing the model in our study with those from other countries, it is found that many of the domains and competencies are similar. Basic medical services, medical knowledge, lifelong learning, interpersonal communication, and teamwork are mentioned in the framework, such as the CanMEDS-FM, WONCA tree, and the Family Medicine Milestones with some different expressions. This may be due to the common nature of GPs worldwide, especially concerning the core competencies required of a qualified GP, irrespective of nationality. However, some differences exist between this Chinese GPs’ model and the others. In China, GPs are required to provide basic public health services. In contrast, public health physicians may carry out this work in the primary healthcare teams or by commercial operations in other countries. Lu et al. developed a competency model for GPs after standardised residency training in China [35]. The research ability was set as a primary indicator, which differed from our study.

The services provided by GPs vary from country to country. In China, GPs primarily work in community and township health centres, diagnosing and treating common diseases, managing chronic diseases, providing health education, and performing other related tasks. However, due to inadequate staffing and poor teamwork, GPs are also expected to undertake public health and management work. Therefore, GPs must possess basic medical and public health services abilities and other competencies outlined in our competency model. In recent years, some secondary and tertiary general hospitals in China have established departments of general practice to teach and train GPs. GPs in these hospitals require specific teaching and research abilities, and scientific research ability is considered a secondary-level item in this general competency model for all GPs.

There are some limitations of the study. During the national survey process, only GPs were investigated. A larger and broader pool of participants with various roles and experiences, such as patients and community residents, could be selected for further validation evidence. It is also important to highlight that the model is dynamic since the nature of any job role changes over time, given the society’s development and changes. Future research should involve re-assessment of the model over time based on such changes and the impact on the job role [36].

## Conclusion

Our study constructed a general competency model for Chinese GPs that was validated to be reliable. The Chinese GPs’ competency framework includes six dimensions: (a) basic medical services, (b) basic public health services, (c) information utilisation ability and management ability, (d) medical knowledge and lifelong learning, (e) interpersonal communication and teamwork and (f) professionalism. This model is consistent with China’s current situation and will provide a good foundation for further training and evaluation of general practitioners in China.

## Data Availability

The datasets used during the current study are available from the corresponding author upon reasonable request.

## Abbreviations

ACGME: Accreditation Council for Graduate Medical Education
AVE: Average of variance extracted
BEIs: Behaviour event interviews
CFA: Confirmatory factor analysis
CFI: Comparative fit index
CFPC: College of Family Physicians of Canada
CR: Composite reliability
CV: Coefficient variations
EFA: Exploratory factor analysis
GMC: General Medical Council
KMO: Kaiser–Meyer–Olkin
NFI: Normed fit index
RCGP: The Royal College of General Practitioners
RMSEA: The root-mean-square error of approximation
